# Meatless Momentum: Food-Related Lifestyle Drivers of Plant-Based Meat Alternative Adoption

**DOI:** 10.3390/nu17223628

**Published:** 2025-11-20

**Authors:** Sylwia Żakowska-Biemans

**Affiliations:** Department of Food Market and Consumer Research, Institute of Human Nutrition Sciences, Warsaw University of Life Sciences—SGGW, Nowoursynowska 159c, 02-776 Warsaw, Poland; sylwia_zakowska_biemans@sggw.edu.pl

**Keywords:** food choice behaviour, sustainable consumption, two-step clustering, lifestyle segmentation, meat reduction

## Abstract

**Background/Objectives**: Interest in alternatives to animal-derived products has gained momentum, driven by health, environmental, and ethical concerns. However, consumer interest in plant-based meat alternatives (PBMAs) remains highly heterogeneous. This study employs the core dimensions of the Food-Related Lifestyle (FRL) framework for consumer segmentation to deepen understanding of PBMA adoption in a meat-centric context such as Poland, and to derive segment-specific recommendations that support the transition toward plant-based diets. **Methods**: A cross-sectional survey was conducted among a representative sample of Polish adults (n = 1200). Consumer segmentation was performed using a two-step cluster analysis based on the three FRL dimensions—food involvement, innovativeness, and responsibility. **Results**: Four distinct consumer clusters emerged, differing significantly across all FRL dimensions (*p* < 0.001). Cluster 1, Traditionalists (18.5%), demonstrated high food involvement but the lowest innovativeness, showing the highest proportion of non-buyers and strong environmental scepticism. Cluster 2, Conscious Food Enthusiasts (24.6%), demonstrated the highest scores across all FRL dimensions, reported the most frequent PBMA purchases, and showed a strong sustainability orientation combined with a pronounced appreciation for sensory quality and eating enjoyment. Cluster 3, Moderates (38.8%) occupied intermediate positions exhibiting moderate PBMA purchasing frequency but expressing concern about food waste. Cluster 4, Careless Food Lovers (18.2%, n = 218), showed high food involvement and innovativeness, but the lowest responsibility, characterised by pronounced environmental scepticism. **Conclusions**: The study shows that consumer segments with similar levels of food involvement differ in their perceptions, beliefs, and willingness to adopt PBMAs, primarily according to their environmental orientation. These findings highlight that a strong interest in food alone does not translate into acceptance of plant-based alternatives unless accompanied by sustainability-oriented values. Overall, the results offer practical guidance for designing marketing, product development, and policy initiatives tailored to distinct consumer profiles, supporting the transition toward more plant-based dietary patterns.

## 1. Introduction

In recent years, the global food system has faced increasing pressure to change due to growing concerns about sustainability, human health and animal welfare. These pressures have intensified research and policy discussions aimed at identifying viable alternatives to conventional meat consumption. A compelling body of research has established that excessive meat consumption significantly contributes to environmental impacts and the prevalence of lifestyle-related diseases [1,2,3,4]. Addressing current health and environmental challenges requires a strategic shift toward reducing meat consumption and increasing reliance on alternative protein sources [5]. There is currently no consensus in the academic or regulatory literature regarding the definition of alternative proteins, as the term encompasses a broad and evolving spectrum of products, ranging from minimally processed plant sources to highly engineered novel proteins [6,7,8,9]. The classification of meat substitutes in the scientific literature varies, but a widely used framework distinguishes three main categories: traditional meat alternatives, new meat-mimicking products, and novel protein-based products [10,11]. The first category includes traditional plant-based foods that do not aim to replicate the sensory characteristics of meat but serve as natural, protein-rich alternatives—such as tofu, tempeh, and seitan. The second category, often referred to in broader literature as plant-based meat alternatives or meat analogues, comprises products designed to imitate the sensory profile of conventional meat, typically made from extruded plant proteins such as soy, pea, or wheat [12]. The third group encompasses novel proteins, including cultured meat and lab-grown meat [13,14], as well as proteins derived from insects, algae, and fungi, which diversify the food supply and help reduce reliance on resource-intensive livestock production [15,16]. 

The trend toward adopting plant-based diets and increasing plant protein intake is primarily driven by growing awareness of the health and environmental benefits of plant-based eating [12,17,18]. In addition, concerns related to animal welfare have gained prominence and are contributing to the shift towards plant-based choices [19,20,21]. Additionally, significant advancements in food technology have played a crucial role in developing a diverse range of innovative and appealing plant-based products, further strengthening the attractiveness of these alternatives [22,23]. 

Although they have gained more recognition and offer potential environmental benefits, plant-based meat alternatives have been criticised for their high level of processing. Indeed, in line with the NOVA food classification system, many plant-based meat alternatives fall into the category of ultra-processed foods (UPFs) [24]. Recent studies indicate that optimised plant-based meat alternatives (PBMAs), although highly processed, have substantial potential to reduce environmental impacts when compared to conventional meat [25,26,27]. Nevertheless, studies consistently show that PBMAs tend to be perceived less favourably than meat [28,29]. There remains considerable scope for improvement in several areas, including taste, texture and price across many product categories [30,31,32]. Consumers have multiple options for adopting a more sustainable, plant-focused diet [3,33]. These include following a vegan or vegetarian lifestyle and reducing meat consumption [34], as well as consuming meat less frequently [33,35]. Addressing these adoption challenges requires an examination of the demographic and psychological profiles of current PBMA consumers, which can reveal important patterns to inform market development strategies. Many studies have shown that consumers of plant-based meat alternatives tend to be younger, predominantly female, have higher levels of education, and express concerns about both their health and the environment [8,36,37,38]. Furthermore, there is evidence that PBMAs are more appealing to individuals with lower levels of attachment to meat [39,40]. Additionally, variations in acceptance levels among different alternative proteins were observed. Studies also report substantial variation in acceptance levels across different alternative protein sources. Plant-based protein options such as pulses receive the highest levels of acceptance, whereas insects are the least accepted and cultured meat is the second least accepted option [41,42]. Pulses and plant-based meat alternatives are also regarded as having higher sustainability potential than many other alternative proteins [23]. This suggests that concentrating efforts on increasing the consumption of pulses and plant-based meat alternatives holds the most promise, both in terms of environmental impact and societal acceptance. Despite the recognised environmental benefits and sensory improvements of these alternatives, their adoption rates remain relatively modest [43,44].

Increasing the adoption of plant-based meat alternatives in everyday diets appears to be a promising approach for promoting more sustainable eating patterns with a smaller environmental footprint [45]. However, given the low intake of these alternatives alongside generally inadequate plant-based food consumption, it is essential to deepen our understanding of the factors that influence such food choices in different cultural contexts, in order to develop effective strategies that encourage consumers to select more diverse protein sources. Previous research on Polish consumers has examined relationships between self-identity and habitual behaviour in eating more plant foods [46], meat consumption and propensity to consume plant products [47], as well as diet quality and past changes in food consumption as determinants of intentions to eat less meat and more plant-based foods [48]. However, a substantial gap remains in comprehensive, theory-driven segmentation analyses that systematically integrate both psychographic and behavioural consumer characteristics to identify segments based on lifestyle factors. To fill this research gap and gain greater insight into the drivers of PBMA consumption among Poles, this study employs the Food-Related Lifestyle (FRL) construct—a theory-driven framework that captures consumers’ cognitive and behavioural patterns related to food purchasing, preparation and consumption. The validity of Food-Related Lifestyle as a basis for segmentation has been demonstrated in international research examining diverse food categories and products with varied attribute profiles [49,50,51,52].

Therefore, the primary objective of this study was to deepen the understanding of the lifestyle factors that influence consumers’ decisions to shift their diets toward plant-based options. The specific aims were to (1) identify distinct consumer segments based on their food-related lifestyle; and (2) characterise these segments according to their self-reported frequency of consuming plant-based alternatives, their perceptions of plant-based meat alternatives, their engagement in sustainability-related behaviours and their sociodemographic characteristics.

## 2. Materials and Methods

### 2.1. Participants

The study was conducted on a nationwide sample applying selection criteria matched to the population distributions in Poland for gender, age, place of residence, and region, using stratified random sampling. Eligibility was restricted to individuals aged 18 years and older who were responsible or co-responsible for decision-making and food purchases within their households. In total, 1200 participants were recruited from an online access panel—a professionally managed database of pre-recruited volunteers for web-based surveys with verified demographic profiles, designed to support population-representative sampling—administered by a market research firm operating in accordance with the ESOMAR International Code on Market, Opinion and Social Research [53] and fully compliant with the EU General Data Protection Regulation (GDPR; Regulation (EU) 2016/679, 27 April 2016 [54]). Panel quality assurance procedures included verification of email addresses, removal of duplicate registrations, monitoring of response patterns to detect fraudulent participants and ongoing panel maintenance to sustain active membership. The required sample size was estimated using Yamane’s formula [55], assuming a population of approximately 38 million adults in Poland, a 95% confidence level and a 3% margin of error, which yielded a minimum sample size of approximately 1100 observations. The achieved sample size therefore exceeded this threshold and was considered adequate for the intended analyses. The study adhered to the ethical principles of the Declaration of Helsinki. Informed consent was obtained prior to data collection. Respondents were informed in advance about the nature of their involvement and their rights, including the right to withdraw from the study at any stage. To begin the questionnaire, participants were required to confirm their understanding of the study’s purpose and indicate their agreement to participate by selecting a consent box. All collected data were encoded in a non-identifiable format and processed anonymously. 

### 2.2. Questionnaire Structure and Measures

The questionnaire consisted of three main sections. The first section addressed food consumption and purchasing behaviours. The second focused on food-related lifestyle aspects, perceptions of PBMAs, beliefs regarding their potential to replace meat and sustainability-oriented behaviours. To ensure a consistent understanding of PBMAs and to minimise interpretative bias, respondents were provided with the following working definition: “Plant-based meat alternatives include pulses such as beans, peas and soy, as well as processed products derived from these, such as tofu, tempeh, seitan and other plant-based protein-rich foods designed to replace meat.” The final section collected information on respondents’ sociodemographic characteristics.

#### 2.2.1. Food-Related Lifestyle Measurement Instrument

For consumer segmentation, the Food-Related Lifestyle framework was used, which conceptualises lifestyle as a cognitive mediator connecting fundamental life values, those ultimate goals people consider important, to how individuals perceive and act towards specific food-related items or situations [50]. This instrument provides a systematic and scientifically well-grounded method for understanding consumers’ food-related lifestyles, which has been validated and applied in cross-cultural research on food and nutrition [56,57,58]. The original version of the FRL consists of 69 statements representing 23 dimensions across five areas: motives linked to food purchases (which can be viewed as domain-specific adaptations of general life values), the type of food quality people seek, how they shop, how they cook, and how they organise their meals [59]. Although numerous international studies have utilised both the full version of FRL and selected domains, the present study employed a new instrument that includes core dimensions of food-related lifestyle, as proposed by Brunsø et al. [50] in 2021. The core dimensions—food involvement and food innovativeness—are directly addressed, and a third dimension, food responsibility, is included to address the recent increased interest in the ethics and sustainability of food. In total, the questionnaire included 15 statements to which participants responded using a 7-point Likert scale, anchored with 1 = completely disagree and 7 = completely agree. The items are presented in the table in Section 3.2.

#### 2.2.2. Characterising Variables

For segment profiling, several measures were utilised. Declared consumption of PBMAs was assessed using the following frequency options: several times a week, once a week, 2–3 times a month, once a month, once every 2–3 months, less than once every three months, or never. Participants’ perceptions of PBMAs were evaluated using a semantic differential scale previously applied in studies on consumer acceptance of alternative protein sources, including insect-based products [60]. Respondents rated their agreement with statements concerning perceived health benefits, environmental impact, nutritional value, protein completeness, taste, availability, ease of preparation, and perceived cost of PBMAs. The reliability of this scale, as measured by Cronbach’s alpha, was acceptable (α = 0.650). To minimise order effects and response bias, items were presented in randomised order across respondents. Beliefs about PBMAs compared to conventional meat were measured as part of the attitudinal section of the questionnaire. The items were developed based on previous studies by Siegrist et al. [61], Michel et al. [61], and Żakowska-Biemans et al. [62,63], reflecting both evaluative and behavioural aspects of attitudes toward PBMAs and meat consumption. Specifically, the statements “Information about the negative environmental impact of eating meat is exaggerated” and “Reducing my meat consumption would not positively impact the environment” were based on Siegrist et al. [61] and demonstrated good internal consistency (Cronbach’s α = 0.803). The items “I try to eat as many plant-based meat substitutes as possible to reduce my meat intake,” “Plant-based meat substitutes replace meat in my kitchen,” and “I try to eat as many plant-protein products as possible (e.g., pulses)” were derived from the Sustainable Healthy Eating scale [62], while “Plant-based meat substitutes should taste like meat” was based on Michel et al. [61] This set of items showed acceptable reliability (Cronbach’s α = 0.738). All items were rated on a 7-point Likert-type scale ranging from “strongly disagree” (1) to “strongly agree” (7). To further examine respondents’ engagement in environmentally and ethically responsible food practices, the questionnaire included selected items that reflected sustainable food consumption behaviours. These items were derived from the Sustainable and Healthy Eating (SHE) scale [62]. Respondents indicated the extent to which each statement applied to their behaviour, including: “I choose free-range eggs,” “I avoid purchasing eggs from caged hens,” “I do not waste food,” “I try to use leftovers to make new meals or snacks,” “I choose foods produced in an environmentally friendly way,” “I choose locally produced food products,” “Whenever possible, I buy fish from sustainable fisheries,” “I try not to throw away food,” and “I limit my meat consumption.” The internal consistency of this scale was satisfactory (Cronbach’s α = 0.824). Finally, the questionnaire collected socio-demographic information, including age, gender, education level, place of residence, income, subjective financial situation, and dietary preferences. The type of diet followed by respondents was determined by the question, “Are you currently following any dietary regime?” with the options: “no special diet,” “vegetarian” (defined as not consuming meat or meat products but including eggs and dairy), “vegan” consuming only plant-based products), or other, with space provided to specify. For further analysis, the “vegetarian” and “vegan” responses were combined into a single group labelled “Vegetarian/Vegan” as these groups individually represented very small proportions of the sample.

### 2.3. Data Analysis

Data were analysed using descriptive statistics, parametric (ANOVA) and non-parametric tests (chi-square), as well as multivariate analysis (factor analysis) and two-step cluster analysis. In the first stage, factor analysis was conducted on FRL using the principal component analysis (PCA) method with varimax rotation. Subsequently, the two-step clustering method was applied to identify consumer segments. Cluster solutions were compared using the Bayesian Information Criterion based on the log-likelihood (BIC-LL) [50]. The BIC-LL reached its lowest value for the four-cluster solution, which was therefore adopted as the final one. After the clusters had been defined, the next step was to profile these segments. Chi-square tests were used to compare socio-demographic characteristics and purchase frequency across clusters. One-way ANOVA was used to test mean differences between clusters for FRL items, perceptions of PBMAs, beliefs, and sustainability-related behaviours. In the tables in Section 3.3, Section 3.5, Section 3.6 and Section 3.7, the reported F statistics correspond to these ANOVA models and test the null hypothesis that the mean value of a given item is equal across all four clusters. The F-statistic represents the ratio of between-cluster variance to within-cluster variance; larger F-values indicate greater differences between clusters relative to the variability within clusters. For each ANOVA, the F-statistic is reported together with its associated degrees of freedom (*df* = 3, 1196) and *p*-value. When the overall F-test was statistically significant, post hoc pairwise comparisons between clusters were conducted using Scheffé tests. In the tables, different superscript letters within a row denote statistically significant differences between cluster means at *p* < 0.05 based on these post hoc tests, whereas asterisks (*, **, ***) indicate the significance level of the overall F test (*p* < 0.05, *p* < 0.01 and *p* < 0.001, respectively). The collected data were analysed using the IBM SPSS Statistics v. 29 statistical software package, Armonk, NY, USA: IBM Corp.

### 2.4. Characteristics of the Sample

The total sample comprised 54.4% female and 45.6% male respondents (the table in Section 3.2). With respect to age, the largest group was aged 50 years or older (45.7%), followed by those aged 35–49 years (23.6%), 25–34 years (18.5%), and 24 years or younger (12.3%). In terms of educational attainment, 41.8% of participants had higher education qualifications, 47.9% had completed secondary education, and 10.3% had primary or vocational education. Regarding employment status, 54.2% were employed, 22.7% were retirees or pensioners, 6.0% were students, and 4.1% were unemployed. Self-reported household financial status was predominantly average (57.3%); 25.3% indicated good conditions, 1.5% very good, and 1.0% very poor. Considering the place of residence, 38.9% of respondents lived in rural areas, while urban respondents were distributed across settlements of varying sizes: 20.8% in towns of 20,000–99,999 residents; 8.4% in cities of 100,000–199,999; 9.3% in cities of 200,000–499,999; and 12.3% in urban areas with more than 500,000 inhabitants.

## 3. Results

### 3.1. Results of Factor Analysis on FRL

The exploratory factor analysis of the core dimensions of FRL, comprising 15 items, identified three principal factors that describe the three core dimensions of food-related lifestyle (Table 1). The Kaiser–Meyer–Olkin (KMO) measure of sampling adequacy was 0.913, indicating that the dataset is suitable for factor analysis. Bartlett’s test of sphericity was significant (*p* < 0.05), confirming sufficient intercorrelations among variables. The analysis produced a three-factor solution that accounted for 67.51% of the total variance. The factor structure obtained in this study reflects that reported by Brunsø et al. [50]. Factor 1 emphasises the enjoyment and social significance of food, highlighting food’s integral role in both daily life and social contexts, corresponding to the involvement dimension of FRL. Factor 2 relates to openness to culinary innovation, characterised by a willingness to explore new tastes and cuisines, aligning with the innovation dimension. Finally, Factor 3, labelled responsibility, focuses on environmental consciousness in food choices, underscoring concerns about the environmental and ethical implications of food production.

### 3.2. Segment Description

The four identified clusters were as follows: Cluster 1 comprised 222 respondents (18.5%), Cluster 2 included 295 respondents (24.6%), Cluster 3 was the largest with 465 respondents (38.8%) and Cluster 4 consisted of 218 respondents (18.2%) (Table 2). Statistically significant differences were found among clusters only for sex (*p* = 0.011) and age (*p* < 0.001), while other socio-demographic factors did not vary significantly across clusters.

Cluster 1 exhibited a balanced gender distribution but showed a higher proportion of respondents aged 50 and above (59.9%), the largest share of retirees (35.6%) and the lowest prevalence of higher education (37.4%). Despite the substantial share of retirees, employment remained common (42.8% in permanent jobs). Respondents in this cluster most frequently assessed their financial situation as average (60.4%) or good (21.2%). This cluster also included a higher proportion of individuals residing in rural areas (41.4% in villages), with limited representation in major metropolitan centres (9.0% in cities with over 500,000 inhabitants).

Cluster 2 was characterised by a predominance of men (62.0%) and a younger age structure (only 35.9% aged 50 and above). The vast majority of respondents in this cluster were employed (58.0%), with a relatively low share of retirees (15.6%). Spatially, this cluster demonstrated the strongest metropolitan orientation (15.3% in cities exceeding 500,000 inhabitants and only 7.8% in towns with up to 20,000 residents). Respondents in this segment most frequently assessed their financial position as good (30.8%), and this cluster had the lowest proportion reporting an average financial situation (49.2%).

Cluster 3 constituted the largest segment and exhibited moderate characteristics across most dimensions. It showed a substantial representation of older adults (50.8% aged 50 and above), combined with the highest educational attainment (44.5% with higher education). The proportion of respondents declaring employment was 54.8%, while retirees constituted 27.3%. The vast majority assessed their financial situation as average (62.6%), and this cluster had the smallest proportion reporting good conditions (21.1%). The residential structure was similar to that of the total sample.

Cluster 4 had a balanced gender distribution, with the highest share of respondents aged 25–34 (28.4%) and the smallest share aged 50 and above (33.5%). It had the highest employment rate (59.6%) and the largest share of students (10.1%), with a low share of retirees (10.1%). In terms of place of residence, the share living in rural areas was lower than in the overall sample (34.9% in villages) and the lowest among all clusters. This cluster also stood out for its substantial presence in large cities, with 15.1% of its members living in cities with more than 500,000 residents. Financial self-assessments indicated that 31.2% reported good conditions and 3.2% very good conditions—the highest proportions across all clusters.

Across the total sample, the vast majority of respondents (90.8%) reported not following any particular diet. Vegetarian or vegan diets were reported by only a small share (3.9%), while 5.3% indicated adherence to another type of diet. Cluster 1 had a high proportion of respondents without a special diet (92.3%), with 3.2% declaring a vegetarian or vegan diet and 4.5% reporting another diet. Cluster 2 showed the greatest dietary diversity, with the lowest share of respondents not following any specific diet (85.8%) and the highest share identifying as vegetarian or vegan (7.5%); an additional 6.8% reported following another type of diet. Cluster 3 largely mirrored the total sample, with 91.6% not following a specific diet, 2.8% declaring a vegetarian or vegan diet and 5.6% reporting another diet. Cluster 4 had the highest proportion of respondents not following any special diet (94.5%) and the lowest shares of respondents who were vegetarian/vegan (2.3%) and those following other diets (3.2%).

**Table 2:** Socio-demographic Characteristics of the Total Sample and by Cluster.

Characteristics	N	(%)	Cluster 1	Cluster 2	Cluster 3	Cluster 4
	1200	100	N = 222	N = 295	N = 465	N = 218
Gender						
Male	547	45.6	(110) 49.5%	(183) 62.0%	(252) 54.2%	(108) 49.5%
Female	653	54.4	(112) 50.5%	(112) 38.0%	(213) 45.8%	(110) 50.5%
Age						
Up to 24	147	12.3	(23) 10.4%	(47) 15.9%	(46) 9.9%	(31) 14.2%
25–34	222	18.5	(29) 13.1%	(57) 19.3%	(74) 15.9%	(62) 28.4%
35–49	283	23.6	(37) 16.7%	(85) 28.8%	(109) 23.4%	(52) 23.9%
Above 50	548	45.7	(133) 59.9%	(106) 35.9%	(236) 50.8%	(73) 33.5%
Education						
Primary	42	3.5	(8) 3.6%	(13) 4.4%	(11) 2.4%	(10) 4.6%
Vocational	82	6.8	(23) 10.4%	(25) 8.5%	(27) 5.8%	(7) 3.2%
Secondary	575	47.9	(108) 48.6%	(136) 46.1%	(220) 47.3%	(111) 50.9%
Higher	501	41.8	(83) 37.4%	(121) 41.0%	(207) 44.5%	(90) 41.3%
Place of residence						
Village/rural	467	38.9	(92) 41.4%	(120) 40.7%	(179) 38.5%	(76) 34.9%
Town up to 20k	120	10.0	(21) 9.5%	(23) 7.8%	(53) 11.4%	(23) 10.6%
City 20–99k	250	20.8	(50) 22.5%	(51) 17.3%	(106) 22.8%	(43) 19.7%
City 100–199k	101	8.4	(19) 8.6%	(32) 10.8%	(30) 6.5%	(20) 9.2%
City 200–499k	112	9.3	(20) 9.0%	(22) 7.5%	(47) 10.1%	(23) 10.6%
City over 500k	148	12.3	(20) 9.0%	(45) 15.3%	(50) 10.8%	(33) 15.1%
Occupation						
Employed (permanent job)	629	54.2	(95) 42.8%	(171) 58.0%	(233) 54.8%	(130) 59.6%
Company co-owner	8	0.7	(2) 0.9%	(3) 1.0%	(3) 0.7%	(0) 0.0%
Self-employed/own business	54	4.7	(7) 3.2%	(17) 5.8%	(17) 4.0%	(13) 6.0%
Student	70	6.0	(11) 5.0%	(18) 6.1%	(19) 4.5%	(22) 10.1%
Casual/odd jobs	66	5.7	(11) 5.0%	(23) 7.8%	(20) 4.7%	(12) 5.5%
On maternity/parental leave	12	1	(0) 0.0%	(4) 1.4%	(4) 0.9%	(4) 1.8%
Retired/on pension	263	22.7	(79) 35.6%	(46) 15.6%	(116) 27.3%	(22) 10.1%
Unemployed	48	4.1	(5) 2.3%	(16) 5.4%	(21) 4.9%	(6) 2.8%
Farm owner/operator	12	1.0	(2) 0.9%	(3) 1.0%	(7) 1.6%	(0) 0.0%
Not working, homemaker	53	4.6	(9) 4.1%	(12) 4.1%	(25) 5.9%	(7) 3.2%
Other situation	6	0.5	(1) 0.5%	(2) 0.7%	(2) 0.5%	(1) 0.5%
Assessment of the financial situation						
We live very poorly—insufficient even for basic needs	12	1	(1) 0.5%	(3) 1.0%	(7) 1.5%	(1) 0.5%
We live modestly—we must economise a lot every day	172	14.3	(38) 17.1%	(48) 16.3%	(62) 13.3%	(24) 11.0%
We live average—enough for daily needs, we must save for bigger purchases	688	57.3	(134) 60.4%	(145) 49.2%	(291) 62.6%	(118) 54.1%
We live well—plenty without special saving	304	25.3	(47) 21.2%	(91) 30.8%	(98) 21.1%	(68) 31.2%
We live very well—we can afford some luxury	18	1.5	(2) 0.9%	(2) 0.7%	(7) 1.5%	(7) 3.2%
Diet						
I do not follow any particular diet	1090	90.8	(205) 92.3%	(253) 85.8%	(426) 91.6%	(206) 94.5%
Vegetarian/Vegan	47	3.9	(7) 3.2%	(22) 7.5%	(13) 2.8%	(5) 2.3%
Other	63	5.3	(10) 4.5%	(20) 6.8%	(26) 5.6%	(7) 3.2%

### 3.3. Food-Related Lifestyle Dimensions Across Consumer Segments

**Figure 1:**
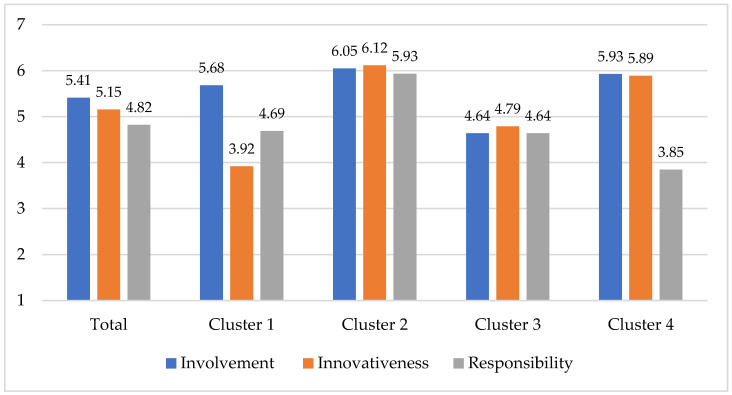
Mean scores for the three core Food-Related Lifestyle dimensions across consumer clusters.

To explore differences in food-related orientations among the identified consumer clusters, mean scores were compared across the three core dimensions of the Food-Related Lifestyle (FRL) framework, namely Involvement, Innovativeness and Responsibility. Figure 1 presents the mean scores for these dimensions.

The one-way ANOVA revealed statistically significant differences between clusters for all three dimensions: Involvement (F(3, 1196) = 380.40, *p* < 0.001), Innovativeness (F(3, 1196) = 413.95, *p* < 0.001) and Responsibility (F(3, 1196) = 324.09, *p* < 0.001). In the total sample, the highest mean was observed for Involvement (5.41), followed by Innovativeness (5.15) and Responsibility (4.82), suggesting that respondents generally displayed stronger emotional engagement with food than concern for ethical or environmental aspects. Comparison of mean scores across clusters revealed distinct differences among consumer groups. Post hoc Scheffé tests confirmed that most inter-cluster differences were statistically significant (*p* < 0.001). For Involvement, Cluster 2 (6.05) and Cluster 4 (5.93) scored significantly higher than Cluster 1 (5.68) and Cluster 3 (4.64), with no significant difference between Cluster 2 and Cluster 4 (*p* = 0.221). For Innovativeness, all clusters differed significantly, with the highest mean observed in Cluster 2 (6.12), followed by Cluster 4 (5.89), Cluster 3 (4.79) and Cluster 1 (3.92). For Responsibility, Cluster 2 (5.93) scored significantly higher than all other clusters—Cluster 1 (4.69), Cluster 3 (4.64) and Cluster 4 (3.85) (all *p* < 0.001)—while no significant difference was found between Cluster 1 and Cluster 3 (*p* = 0.892).

To provide a more detailed view of how the general lifestyle dimensions are reflected in specific attitudes and behaviours, Table 3 presents the mean scores for individual FRL items across the consumer clusters, along with the corresponding ANOVA statistics. The analysis revealed clear and statistically significant differences among clusters across all FRL items (all *p* < 0.001). In the total sample, the highest mean scores were recorded for “I just love good food” (5.64) and “Food and drink are an important part of my life” (5.50). Conversely, items related to environmental and ethical considerations received considerably lower ratings, such as “I try to buy organically produced foods if possible” (4.43) and “I try to choose food that is produced in a sustainable way” (4.96).

Based on the differences observed in food-related lifestyle dimensions and respondents’ profiles, the following labels were proposed for the identified segments: Cluster 1—Traditionalists, Cluster 2—Conscious Food Enthusiasts, Cluster 3—Moderates, and Cluster 4—Careless Food Lovers.

Overall, the largest inter-cluster differences were found for items related to culinary innovativeness, particularly “I love to try recipes from different countries” (F(3, 1196) = 285.10, *p* < 0.001), “I like to try new foods that I have never tasted before” (F(3, 1196) = 246.84, *p* < 0.001) and “I like to try out new recipes” (F(3, 1196) = 250.79, *p* < 0.001), as well as for environmental responsibility, for example “I try to choose food produced with minimal impact on the environment” (F(3, 1196) = 209.99, *p* < 0.001). Cluster 2 consistently scored highest across most items, especially those indicating innovativeness and responsibility, such as “I like to try new foods that I have never tasted before” and “I try to choose food produced with minimal impact on the environment”. Cluster 4 also displayed high involvement and enjoyment in food-related experiences and scored highly on items reflecting culinary exploration such as “I just love good food” and “I like to try out new recipes”, but recorded the lowest mean scores on responsibility-related items, including “I try to choose food produced with minimal impact on the environment” and “I try to buy organically produced foods if possible”. Cluster 3 reported the lowest mean values on items capturing the importance of food in everyday and social life, such as “Food and drink are an important part of my life” and “Eating and food is an important part of my social life”, reflecting lower overall food involvement. In contrast, Cluster 1 exhibited moderate food involvement but scored lowest on several items related to culinary innovativeness, such as “I like to try new foods that I have never tasted before” and “I love to try recipes from different countries”, and showed lower environmental responsibility than Cluster 2, although higher than Cluster 4.

### 3.4. Frequency of Purchasing and Consuming PBMA

Across the total sample, 14.3% of respondents reported purchasing PBMA once a week or more, 20.1% two to three times a month, 17.2% once a month, 10.4% every two to three months, and 13.8% about once every three months, while 24.2% declared that they do not buy them at all (Table 4). 

Cluster membership was significantly associated with PBMA purchasing frequency (χ^2^ = 130.588, *df* = 15, *p* < 0.001). Cluster 1 (Traditionalists) comprised the highest share of non-buyers (38.3%) and showed the lowest frequency of regular purchasing, with only 9.9% buying PBMAs weekly or more often and 11.3% buying them two to three times a month. Cluster 2 (Conscious Food Enthusiasts) was characterised by the most frequent PBMA purchasing behaviour. More than half of the respondents in this group reported frequent buying, with 26.4% purchasing PBMAs weekly or more often and 28.5% two to three times a month. Importantly, only 11.5% reported never having bought PBMAs, which represents the lowest share among all clusters. Cluster 3 (Moderates) demonstrated regular but less frequent purchasing patterns. The largest share of respondents in this cluster reported medium-frequency buying, with 18.5% purchasing PBMAs two to three times a month and 22.2% about once a month, while 22.2% indicated that they never buy PBMAs. Cluster 4 (Careless Food Lovers) exhibited a more heterogeneous pattern. Although 14.7% reported purchasing PBMAs weekly or more often and 21.1% two to three times a month, a relatively large proportion (31.2%) stated that they do not buy PBMAs at all.

### 3.5. Perceptions of Plant-Based Meat Alternatives Across Consumer Segments

The perception of PBMA attributes reveals distinct patterns across the entire sample and significant differences between consumer clusters (Table 5). The respondents demonstrated the most positive perceptions regarding environmental benefits (5.26), followed by healthfulness (5.00) and protein completeness (4.59), while taste was scored the lowest (3.55) across the total sample.

The analysis identified taste perception and nutritional value as the attributes that most strongly differentiated the consumer clusters. Cluster 2 (Conscious Food Enthusiasts) consistently demonstrated the most favourable perceptions of PBMAs, rating them significantly higher than the other groups in terms of healthfulness, environmental benefits, nutritional value, protein completeness, availability and ease of preparation. Cluster 1 (Traditionalists) expressed more sceptical attitudes, providing moderate evaluations of healthfulness and environmental benefits, but significantly lower scores for nutritional value and protein completeness compared with Cluster 2. Cluster 3 (Moderates) generally occupied an intermediate position, with evaluations close to the sample mean, whereas Cluster 4 (Careless Food Lovers) exhibited the most critical attitudes, assigning the lowest scores across nearly all attributes, particularly in terms of taste, nutritional value and environmental benefits. The strongest inter-cluster differences were observed for taste (F(3, 1196) = 28.35, *p* < 0.001), nutritional value (F(3, 1196) = 24.07, *p* < 0.001) and environmental benefits (F(3, 1196) = 12.87, *p* < 0.001). The “special meal versus everyday” dimension showed no significant variation, indicating generally consistent perceptions of PBMA’s role in daily eating patterns across clusters.

### 3.6. Cluster-Specific Beliefs, and Behaviours Regarding Plant-Based Meat Alternatives Versus Meat

Characterising consumer segments based on their environmental perceptions, taste expectations and willingness to reduce meat consumption helps to capture the underlying drivers and barriers to the adoption of PBMAs. In the overall sample, opinions on introducing plant-based options as meat substitutes revealed a mixed pattern. The mean score for “I try to eat as many plant-based meat substitutes as possible to reduce my meat intake” was relatively low (3.59), and even lower for “Plant-based meat substitutes replace meat in my kitchen” (3.31) (Table 6). In contrast, more general plant-protein sources, such as legumes, were evaluated more positively (4.16). The highest mean score in the total sample was observed for “Plant-based meat substitutes should taste like meat” (4.48), underscoring the importance of taste expectations.

Cluster 1 (Traditionalists) showed relatively high agreement with statements expressing scepticism toward the environmental benefits of meat reduction, although their scores were lower than those of Cluster 4. The mean score was 4.50 for “Information about the negative environmental impact of eating meat is exaggerated” and 4.32 for “Reducing my meat consumption would not positively impact the environment” (F(3, 1196) = 21.62 and F(3, 1196) = 15.68, both *p* < 0.001). 

Cluster 2 (Conscious Food Enthusiasts) reported significantly higher mean scores than all other clusters on items promoting plant-based consumption and meat reduction. The mean score for “I try to eat as many plant-protein products as possible (e.g., pulses)” was 5.03 and 4.59 for “I try to eat as many plant-based meat substitutes as possible to reduce my meat intake” (F(3, 1196) = 61.20 and F(3, 1196) = 81.58, both *p* < 0.001). 

Cluster 3 (Moderates) generally remained close to the overall average across most statements, with mean scores of 4.31 for “Plant-based meat substitutes should taste like meat” and 4.09 for “I try to eat as many plant-protein products as possible (e.g., pulses)”. Lower scores were observed in this cluster for statements directly related to meat replacement behaviour, such as “Plant-based meat substitutes replace meat in my kitchen” (3.38; F(3, 1196) = 77.55, *p* < 0.001). 

Cluster 4 (Careless Food Lovers) recorded significantly lower scores on items related to PBMA consumption and meat reduction, while simultaneously showing the highest scepticism regarding environmental benefits. The mean score for “Plant-based meat substitutes replace meat in my kitchen” was 2.42 and 2.59 for “I try to eat as many plant-based meat substitutes as possible to reduce my meat intake” (F(3, 1196) = 77.55 and F(3, 1196) = 81.58, both *p* < 0.001). In contrast, “Plant-based meat substitutes should taste like meat” received a relatively higher mean score of 4.22 in this cluster (F(3, 1196) = 11.85, *p* < 0.001).

### 3.7. Sustainable and Ethical Food Consumption Behaviours Across Consumer Segments

To gain deeper insight into respondents’ willingness to engage in environmental and ethical food-related practices and to expand the food responsibility dimension newly added to the Food-Related Lifestyle framework, respondents were asked about selected behaviours reflecting the idea of sustainable food consumption. The overall results indicate relatively high commitment to food waste prevention, as shown by the very high mean scores for “I try not to throw away food” (6.16) and “I do not waste food” (6.00) (Table 7). Engagement in using leftovers was also relatively high (5.50), confirming that waste avoidance is a widely shared practice. Ethical choices related to animal welfare were less pronounced at the aggregate level. More moderate scores were recorded for environmentally oriented behaviours, including choosing foods produced in an environmentally friendly way (4.77), selecting locally produced food products (4.88) and purchasing fish from sustainable fisheries whenever possible (4.47).

The results indicate statistically significant differences among clusters for all items (*p* < 0.01, with most at *p* < 0.001), pointing to considerable variation in the extent to which consumers engage in sustainability-related practices. The largest inter-cluster differences were observed for choosing foods produced in an environmentally friendly way (F(3, 1196) = 91.16, *p* < 0.001) and limiting meat consumption (F(3, 1196) = 68.61, *p* < 0.001).

Cluster 1 (Traditionalists) showed moderate engagement in sustainable and ethical food behaviours. Members of this group scored relatively high on waste prevention (“I try not to throw away food” = 6.25; “I do not waste food” = 5.99) but exhibited lower involvement in animal-welfare-related actions than Cluster 2, for example “I avoid purchasing eggs from caged hens” (4.83; F(3, 1196) = 22.89, *p* < 0.001) and “I choose free-range eggs” (5.44; F(3, 1196) = 24.70, *p* < 0.001). They also reported limited engagement in environmentally motivated purchasing, as indicated by “I choose foods produced in an environmentally friendly way” (4.58; F(3, 1196) = 91.16, *p* < 0.001) and “I choose locally produced food products” (4.75; F(3, 1196) = 40.52, *p* < 0.001).

Cluster 2 (Conscious Food Enthusiasts) demonstrated the highest overall involvement, scoring significantly higher than the other groups on nearly all items. Members of this cluster reported strong engagement in ethical and environmentally responsible consumption, such as “I choose free-range eggs” (6.13) and “I avoid purchasing eggs from caged hens” (5.65), reflecting a pronounced sensitivity to animal welfare concerns. They also showed strong commitment to supporting local and eco-friendly production, including “I choose locally produced food products” (5.58) and “I choose foods produced in an environmentally friendly way” (5.74), as well as higher engagement in limiting meat consumption (4.87).

Cluster 3 (Moderates) generally occupied an intermediate position, with scores close to the overall mean across most behaviours. For example, mean scores were 5.97 for “I try not to throw away food”, 5.37 for “I try to use leftovers to make new meals or snacks” and 4.77 for “I choose locally produced food products”. Although they reported somewhat higher engagement in limiting meat consumption (4.21) compared with Cluster 1 and Cluster 4, their involvement in animal-welfare and environmentally oriented purchasing remained lower than in Cluster 2.

Cluster 4 (Careless Food Lovers) also displayed high engagement in food waste prevention (“I try not to throw away food” = 6.23; “I do not waste food” = 6.10), but scored significantly lower on meat reduction (2.71; F(3, 1196) = 68.61, *p* < 0.001) and on most behaviours related to animal welfare and environmental sustainability, such as “I avoid purchasing eggs from caged hens” (4.33), “I choose foods produced in an environmentally friendly way” (3.88) and “Whenever possible, I buy fish from sustainable fisheries” (3.86). Overall, Cluster 2 stands out as the most engaged in sustainable and ethical food practices, whereas Cluster 4 shows the weakest commitment beyond food waste prevention. The overview of the cluster characteristics is presented in Table 8.

## 4. Discussion

The present study utilised the three core dimensions of the 15-item Food-Related Lifestyle instrument with the aim of applying it in consumer segmentation to better understand the complex interplay between lifestyle and the propensity to consume PBMAs. The three-factor structure obtained through factor analysis—comprising food involvement, food innovativeness, and food responsibility—aligns with the framework proposed by Brunsø et al. [50], who emphasised that these dimensions provide a short yet meaningful basis for segmenting food consumers in a cross-cultural context. The analysis confirmed that Polish consumers’ responses reflect distinct orientations toward the enjoyment and social importance of food (Involvement), openness to new food experiences (Innovativeness), and concern for environmental and ethical aspects of food choices (Responsibility), consistent with previous validations of the FRL instrument [50]. Overall, respondents demonstrated relatively high food involvement, moderate scores on food innovativeness, and lower scores on food responsibility (environmental and ethical concerns). This pattern suggests that, while enjoyment of food is central, sustainability-oriented motives are less pronounced on average, consistent with recent cross-cultural studies indicating that sensory pleasure and hedonic motivations remain primary drivers of food choices across different cultural contexts [64,65]. This finding aligns with contemporary research demonstrating that pleasure-seeking motives continue to outweigh environmental concerns in food-related decisions, even as awareness of sustainability increases [66]. 

### 4.1. Consumer Segment Profiles and Lifestyle Differentiation

Building on the core FRL dimensions, a cluster analysis revealed four distinct consumer segments differing in their food-related lifestyles but also in their PBMA purchasing frequency, perceptions and intentions regarding PBMA consumption, inclination to reduce meat consumption, beliefs about the environmental impact of meat, and broader sustainability-related behaviours. These segments—Traditionalists, Conscious Food Enthusiasts, Moderates, and Careless Food Lovers—represent distinct consumer profiles.

Traditionalists (Cluster 1) are predominantly older individuals who attribute considerable importance to food in their daily lives but exhibit relatively low openness to new food products. This pattern suggests substantial resistance to PBMAs, likely shaped by dietary conservatism and comparatively weaker perceived sensory appeal, in line with previous findings [41,42]. This segment exhibits the lowest level of engagement with PBMA, with more than one-third of respondents reporting that they have never purchased such products. This cluster’s reluctance to adopt plant-based alternatives underscores the challenges facing PBMA penetration in consumer segments deeply rooted in traditional eating habits. Taste concerns represent the most significant barrier to PBMA adoption across resistant consumer segments [67,68]. Contemporary research consistently identifies sensory attributes as the main barrier, with an unpleasant taste ranking among the three most common obstacles to plant-based protein consumption [69]. Consumers often perceive PBMAs as lacking the “authentic” flavour, juiciness, and umami of traditional meat [61,70]. This sensory scepticism is reinforced by texture and aroma deficiencies, which reduce hedonic satisfaction and overall willingness to purchase [71,72]. Such sensory barriers are particularly pronounced among Traditional consumer segments who maintain strong attachments to meat products and established culinary practices [43,73]. The persistence of negative taste expectations reflects deeper psychological barriers rooted in familiarity preferences and cultural food traditions [74,75]. Recent findings suggest that sensory re-education and informed tasting can reduce bias and improve consumer openness toward PBMA products [76]. Another factor underlying the lower frequency of PBMA consumption among Traditionalists is the attachment to meat, which reflects the perception of meat’s central role in the diet, particularly in meat-centric cultures such as Poland [40]. Environmental scepticism regarding the benefits of meat reduction constitutes a significant cognitive barrier that transcends product-specific attributes. This resistance is anchored in cognitive dissonance, constrained information processing, prevailing social norms, and the systematic avoidance of information that challenges established beliefs [77,78]. 

Compared with Traditionalists (Cluster 1), Conscious Food Enthusiasts (Cluster 2) score highest across all three FRL dimensions, including openness to novelty. They strongly value food enjoyment, explore new cuisines, and scored the highest on environmental and ethical considerations. As a result, they represent the most frequent consumers of PBMAs, with the majority purchasing these products at least monthly and many on a weekly basis. The results indicate that the Conscious Food Enthusiasts (Cluster 2), characterised by pro-environmental orientations, constitute the group most receptive to incorporating this product category into their diets. Their profile suggests that they may act as early adopters and potential opinion leaders in driving the broader acceptance of PBMAs. Conscious Food Enthusiasts demonstrate a strong inclination towards sustainability, echoing the growing body of research that links pro-environmental attitudes to sustainable food choices [79,80]. Previous research consistently demonstrates that perceived environmental and health benefits are key predictors of consumer acceptance of meat alternatives [61,81]. The high frequency of PBMA purchases observed in this group indicates readiness to incorporate such products into everyday eating routines, supporting findings that pro-environmental and health-oriented attitudes can translate into actual consumption behaviour [82]. Empirical studies further show that consumers who are open to plant-based products often share psychographic traits with broader sustainability-conscious and health-oriented segments [71]. Moreover, cross-cultural evidence indicates that individuals motivated by sustainability also tend to engage in dietary experimentation, viewing PBMAs as a means of aligning taste enjoyment with moral and environmental responsibility [81,83,84]. Simultaneously, Cluster 2 represents a consumer segment with high expectations related to sensory attributes of PBMA, as reflected in their high mean scores for statements indicating that plant-based meat alternatives should taste like meat. The emphasis on making PBMA taste like meat provides a clear direction for innovation but also risks reinforcing the development of ultra-processed products, a trend increasingly criticised in public health discourse [85,86]. Given that the inferior sensory characteristics of plant-based meat alternatives, compared to conventional meat products, remain a significant barrier to consumer adoption [17,40], these findings indicate that sensory limitations should not only be addressed at the product development level, but also when developing communication strategies and engaging with highly food-involved consumer segments.

Moderates (Cluster 3) occupy an intermediate position, exhibiting average levels of food involvement, moderate curiosity about new foods, and moderate concerns about sustainability. Their engagement with PBMAs is occasional rather than regular, and a substantial share still never buy these products. Cluster 3 (Moderates), seems to be influenced by both an openness to new experiences and an existing attachment to traditional dietary preferences. For PBMA to reach this segment, strategies that reduce perceived risk and enhance familiarity, such as product sampling and the use of familiar flavour profiles, could be effective [41]. The clear tendency to avoid wasting food among Moderates provides a valuable entry point for fostering broader sustainable food behaviours due to the psychological spillover effect [87,88]. Evidence shows that individuals who actively engage in food waste reduction are more likely to adopt other pro-environmental behaviours [89,90]. This group’s commitment to minimising waste reflects a pre-existing cognitive framework oriented toward resource efficiency, creating favourable conditions for introducing PBMAs as a natural extension of their sustainability practices. This combination presents an opportunity to frame PBMAs’ consumption within narratives that emphasise resource efficiency and waste prevention rather than abstract environmental goals [90].

Careless Food Lovers (Cluster 4) form a distinct group that shares with Cluster 2 a high level of food involvement and culinary adventurousness, reflecting a strong interest in taste experiences and experimenting with new recipes. However, they differ significantly in food responsibility, scoring low on items on sustainability concerns, with many members purchasing PBMAs rarely or avoiding them altogether. This segment enjoys food for pleasure’s sake and novelty, yet remains largely indifferent to environmental or ethical issues. Their stance toward PBMA is predominantly critical; they find these products comparatively less tasty, doubt their health value, and are least convinced about their environmental benefits. Many in Cluster 4 either rarely purchase meat alternatives, and this group displayed the least willingness to reduce meat consumption. Careless Food Lovers, much like Traditionalists, demonstrated scepticism toward the environmental rationale for meat reduction. For example, Cluster 4 respondents tended to agree that claims about the environmental harm of meat are exaggerated and expressed doubt that reducing meat intake would yield environmental benefits. These findings are consistent with previous research identifying a segment of consumers characterised by limited awareness of the environmental impacts of meat production and low concern for sustainability in their dietary choices [91]. Prior literature also suggests that while pro-environmental attitudes can influence acceptance of meat alternatives, environmental concern alone may not be sufficient to induce a dietary shift if the sensory and economic expectations are not met [84,92]. Our data support this finding: the segment most receptive to PBMA (Cluster 2) was one that combined high sustainability values and high culinary involvement, indicating that they were motivated by both ethics and health, as well as open to novel food experiences. Moreover, Cluster 4, in contrast to Cluster 2, scored significantly lower on items related to animal welfare concerns and the importance of sourcing fish sustainably. Concerns about animal welfare constitute another important food attribute differentiating approaches to PBMA [92,93,94]. Members of Cluster 4 value food novelty, but lack a motive to compromise on meat enjoyment for the sake of the environment—and accordingly, they have not embraced PBMA. This interaction between motives is echoed by international studies, which show that consumers generally expect no sacrifice in taste (or cost) even if they recognise the environmental benefits of plant-based foods [68]. 

### 4.2. Interpreting Consumer Segments Through the COM-B Model of Behaviour Change

The four consumer clusters derived from food-related lifestyle segmentation display distinct adoption patterns that can be explained through the COM-B model of behaviour change (Capability, Opportunity, Motivation) [95,96], which assumes behaviour occurs when individuals have the necessary capability, encounter sufficient opportunity in their environment, and possess the motivation to act. In our sample, most respondents showed adequate capability, as basic cooking skills and everyday food management were common; however, limited experience with plant-based meat alternatives restricted their practical use. This finding aligns with Finnish research, where unfamiliarity with pulses and PBMA was one of the most frequently reported barriers, particularly among individuals with lower levels of education or experiencing financial strain [67]. This gap was interpreted as psychological capability—a lack of know-how on how to incorporate these products into meals. 

Although PBMA availability has improved, perceived barriers related to price, convenience, and especially taste reduce the perception that these products are genuinely accessible. Social opportunity, evident in cultural norms favouring meat, also hampers regular adoption. The main difference across segments lies in motivation, understood both as reflective drivers—such as values and environmental responsibility—and as automatic drivers—such as hedonic reward. Within this framework, Traditionalists display adequate capability but limited motivation, particularly on the reflective side, due to environmental scepticism and a cultural attachment to meat. Their social opportunities are limited by traditional norms. 

Conscious Food Enthusiasts combine high capability, strong urban opportunities, and both reflective and automatic motivations, leading to frequent use of plant-based meat alternatives. Moderates present a mixed profile. Capability and motivation are moderate, but their strong concern about food waste provides a valuable motivational anchor that can be leveraged to promote these products within a broader sustainability narrative.

Careless Food Lovers demonstrate high capability and good opportunities, but weak reflective motivation. Their choices are primarily driven by taste and enjoyment, resulting in less engagement with environmental or ethical considerations. For Traditionalists, strengthening capabilities through simple substitution recipes and restructuring opportunities, facilitated by increased visibility and affordability, can help facilitate initial engagement. 

For Conscious Food Enthusiasts, reinforcing reflective motivation with clear sustainability information and social modelling can sustain and expand behaviour. For Moderates, linking plant-based options to food waste reduction and everyday practicality can enhance motivation. For Careless Food Lovers, strategies targeting automatic motivation by emphasising sensory pleasure, novelty, and convenience are more likely to succeed than moral or environmental appeals. Overall, applying the COM-B framework suggests that adoption in Poland is mainly limited by motivation and opportunity rather than capability; therefore, behaviour change initiatives should focus on improving the product experience, especially in terms of taste and convenience, while addressing the diverse motivational profiles observed across segments.

### 4.3. Managerial and Policy Implications

Beyond their theoretical contributions, the findings of this study also offer clear managerial implications for industry stakeholders, policymakers, and communication strategists aiming to accelerate the adoption of PBMA. The segmentation results provide clear strategic directions for industry stakeholders and policymakers seeking to accelerate PBMA adoption. For product development, manufacturers should prioritise sensory improvement and taste enhancement, particularly targeting the perceived taste–sustainability trade-off that influences adoption decisions across all segments. Marketing and communication strategies should be tailored to suit cluster-specific motivations. For Cluster 2 (Conscious Food Enthusiasts), campaigns should highlight environmental benefits, sustainability credentials, and ethical values. Cluster 4 (Careless Food Lovers) requires a different approach focused on taste, convenience, and culinary enjoyment rather than environmental appeals, which might provoke resistance. For the more moderate Clusters 1 and 3, balanced messaging combining practical benefits with gradual awareness of environmental issues is likely to be most effective. A common insight across the segmentation is the shared concern about food waste, presenting a unifying communication opportunity across all segments. Regardless of lifestyle orientation, consumers generally support waste reduction, providing PBMA producers with a credible narrative for collective engagement. This can be reinforced by emphasising how plant-based production aligns with circular economy principles, transforming often by-products into protein sources. 

### 4.4. Limitations and Directions for Future Research

Before drawing final conclusions, several limitations of this study should be acknowledged. First, the sample was drawn from an online panel, which, although stratified to reflect national demographics, may underrepresent individuals without internet access or those less inclined to participate in surveys. This may introduce selection bias, favouring more educated or engaged consumers. Second, all data were self-reported, raising concerns about accuracy and potential social desirability effects. Third, the study employed a cross-sectional design, capturing data at a single point in time. While the findings suggest that lifestyle orientation influences PBMA usage, the reverse relationship is also possible—exposure to or experience with PBMAs may, in turn, shape consumer attitudes. For example, an unfavourable sensory experience could reinforce conservative preferences among Traditionalist consumers. Moreover, the study was conducted in a single cultural context—Poland, a country with a strongly meat-centric food culture. While this focus yields valuable insights into Polish consumers, the generalizability of the findings to other cultures may be limited. Furthermore, the study did not examine the influence of social factors on PBMA adoption. Social influence—including the roles of family habits, peer norms, and media exposure—has been shown to significantly shape food-related behaviours and perceptions of sustainability. Building on these results, several avenues for future research emerge. Cross-cultural studies would be especially valuable: applying this segmentation framework in other countries, or across broader European samples, could reveal whether similar consumer clusters exist elsewhere and how cultural contexts shape their motivations. Such work could guide international PBMA marketing strategies and inform regionally tailored public policy. Future research should also consider intervention studies explicitly grounded in the COM-B framework to identify which behavioural components—building capability through cooking confidence, expanding opportunity through visibility and affordability, or reinforcing motivation through ethical incentives and sensory rewards—most effectively promote plant-based choices. In addition, behavioural nudging approaches could be explored to test subtle contextual interventions that make plant-based selections easier and more attractive. Adjusting default options, redesigning product placement, or integrating social cues into food environments may help trigger both motivation and opportunity simultaneously. Combining experimental and field-based studies would provide robust evidence on how these mechanisms operate in real-life settings. Addressing these areas would contribute to a more comprehensive understanding of how to foster dietary transitions at both the individual and societal levels.

## 5. Conclusions

Considering today’s environmental challenges, there is an urgent need to shift dietary behaviours towards more sustainable and plant-based patterns, especially among those in meat-centric food cultures. The success of initiatives aimed at reducing meat consumption depends on both expanding the availability of meat substitutes that gain consumer acceptance and developing communication strategies that address consumer predispositions and barriers to adopting plant-based meat alternatives. This study contributes to a better understanding of the complex interplay between consumer lifestyles and the adoption of PBMAs by applying the FRL framework. This integration offers a more nuanced view of how enduring lifestyle values influence consumers’ openness to dietary innovation and their engagement with sustainability-oriented food choices. The findings suggest that the importance consumers attach to different lifestyle dimensions is associated with their perceptions of PBMAs and their willingness to adopt them, thereby facilitating a more diverse range of protein sources and potentially lower meat consumption. The contrast between Cluster 2 (Conscious Food Enthusiasts) and Cluster 4 (Careless Food Lovers)—both highly food-involved—illustrates how food involvement can lead consumers in opposite directions depending on their level of food responsibility. The results indicate that PBMA consumption is closely linked to environmental values and concerns about animal welfare, yet they also reveal a segment characterised by strong food involvement but resistance to environmentally oriented choices, underscoring the need for tailored communication approaches. Highlighting the contribution of PBMAs to environmental sustainability and encouraging more ethical food choices remain important components of communication strategies. However, the findings also show that addressing sensory perceptions is equally critical, as many consumers perceive trade-offs in taste and do not wish to sacrifice sensory enjoyment. Each segment’s unique characteristics offer opportunities for targeted marketing, product innovation, and policy initiatives that could collectively accelerate the transition towards more sustainable dietary practices.

## Figures and Tables

**Table 1:** The result of factor analysis on the core dimension of the Food-related lifestyle measurement instrument.

Core Dimensions of Food Related Lifestyle	Involvement	Innovativeness	Responsibility
I just love good food	0.779		
Eating and drinking are a continuous source of joy for me.	0.812		
Decisions on what to eat and drink are very important to me.	0.595		
Food and drink is an important part of my life.	0.840		
Eating and food is an important part of my social life.	0.715		
I like to try new foods that I have never tasted before.		0.818	
I love to try recipes from different countries.		0.831	
Recipes and articles on food from other culinary traditions encourage me to experiment in the kitchen.		0.827	
I like to try out new recipes.		0.852	
I look for ways to prepare unusual meals.		0.697	
I try to choose food produced with minimal impact on the environment.			0.829
I am concerned about the conditions under which the food I buy is produced.			0.703
It is important to understand the environmental impact of our eating habits.			0.755
I try to choose food that is produced in a sustainable way.			0.882
I try to buy organically produced foods if possible.			0.746

**Table 3:** Consumer Segments Defined by Food-Related Lifestyle Dimensions.

Items Representing the Core Dimension of the Food-Related Lifestyle	Total	Cluster 1	Cluster 2	Cluster 3	Cluster 4	F-Value _(3, 1196)_
I just love good food	5.64	5.86 ^b^	6.21 ^c^	4.83 ^a^	6.36 ^c^	224.414 ***
Eating and drinking are a continuous source of joy for me	5.28	5.57 ^b^	5.95 ^c^	4.43 ^a^	5.91 ^c^	209.72 ***
Decisions on what to eat and drink are very important to me	5.59	5.86 ^b^	6.26 ^c^	5.01 ^a^	5.68 ^b^	145.525 ***
Food and drink is an important part of my life	5.50	5.96 ^b^	6.04 ^b^	4.65 ^a^	6.13 ^b^	228.874 ***
Eating and food is an important part of my social life	5.04	5.16 ^b^	5.78 ^c^	4.28 ^a^	5.56 ^c^	135.177 ***
I like to try new foods that I have never tasted before	5.32	4.04 ^a^	6.15 ^c^	4.97 ^b^	6.22 ^c^	246.838 ***
I love to try recipes from different countries	5.00	3.56 ^a^	6.07 ^c^	4.62 ^b^	5.86 ^c^	285.101 ***
Recipes and articles on food from other culinary traditions encourage me to experiment in the kitchen	4.98	3.58 ^a^	6.03 ^d^	4.63 ^b^	5.71 ^c^	268.588 ***
I like to try out new recipes	5.36	4.14 ^a^	6.27 ^c^	5.02 ^b^	6.07 ^c^	250.789 ***
I look for ways to prepare unusual meals	5.12	4.28 ^a^	6.05 ^d^	4.70 ^b^	5.60 ^c^	177.91 ***
I try to choose food produced with minimal impact on the environment	4.79	4.60 ^b^	5.99 ^c^	4.57 ^b^	3.75 ^a^	209.989 ***
I am concerned about the conditions under which the food I buy is produced	4.77	4.73 ^b^	5.79 ^c^	4.60 ^b^	3.78 ^a^	128.064 ***
It is important to understand the environmental impact of our eating habits	5.16	5.17 ^b^	6.18 ^c^	4.92 ^b^	4.29 ^a^	140.776 ***
I try to choose food that is produced in a sustainable way	4.96	4.83 ^b^	6.06 ^c^	4.74 ^b^	4.06 ^a^	176.981 ***
I try to buy organically produced foods if possible	4.43	4.05 ^b^	5.64 ^d^	4.36 ^c^	3.34 ^a^	152.282 ***

Values represent mean scores on a 7-point scale, anchored at “totally disagree” and “totally agree”. Different superscripts within rows indicate statistically significant differences between groups at *p* < 0.05 based on one-way ANOVA with Scheffé post hoc tests. Significant differences between clusters are indicated at the 0.001 levels, denoted by ***.

**Table 4:** Buying frequency of PBMA per cluster.

Purchase Frequency	Total	Cluster 1	Cluster 2	Cluster 3	Cluster 4
Once a week or more often	(172) 14.3%	(22) 9.9%	(78) 26.4%	(40) 8.6%	(32) 14.7%
Two to three times a month	(241) 20.1%	(25) 11.3%	(84) 28.5%	(86) 18.5%	(46) 21.1%
Once a month	(207) 17.2%	(30) 13.5%	(46) 15.6%	(103) 22.2%	(28) 12.8%
Once every 2–3 months	(125) 10.4%	(23) 10.4%	(24) 8.1%	(55) 11.8%	(23) 10.6%
About once every three months	(165) 13.8%	(37) 16.7%	(29) 9.8%	(78) 16.8%	(21) 9.6%
I do not buy them at all	(290) 24.2%	(85) 38.3%	(34) 11.5%	(103) 22.2%	(68) 31.2%

**Table 5:** Perception of selected attributes of PBMA per cluster.

Attribute	Total	Cluster 1	Cluster 2	Cluster 3	Cluster 4	F-Value _(3, 1196)_
Unhealthful—Healthful	5.00	4.96 ^a^	5.48 ^b^	4.82 ^a^	4.79 ^a^	10.646 ***
Harmful for the environment—Beneficial	5.26	5.21 ^a^	5.70 ^b^	5.16 ^a^	4.91 ^a^	12.867 ***
Cheap—Expensive	4.65	4.81 ^ab^	4.44 ^b^	4.54 ^b^	5.02 ^a^	6.708 ***
Low nutritional value—High	4.44	4.06 ^a^	5.02 ^c^	4.48 ^b^	3.95 ^a^	24.067 ***
Special meal—Every day	4.45	4.35 ^a^	4.66 ^a^	4.41 ^a^	4.33 ^a^	6.405
Low availability—High	4.37	4.34 ^ab^	4.73 ^b^	4.30 ^a^	4.10 ^a^	6.697 ***
Difficult to prepare—Easy	4.37	4.23 ^a^	4.73 ^b^	4.32 ^a^	4.15 ^a^	2.636 *
Not tasty—Tasty	3.55	3.96 ^ab^	5.07 ^c^	4.35 ^b^	3.79 ^a^	28.52 ***
Incomplete protein—Complete	4.59	4.48 ^ab^	5.03 ^c^	4.59 ^b^	4.13 ^a^	12.545 ***

Values represent mean scores on a 7-point semantic differential scale, anchored at “does not apply at all” and “definitely applies”. Different superscripts within rows indicate statistically significant differences between groups at *p* < 0.05 based on one-way ANOVA with Scheffé post hoc tests. Significant differences between clusters are indicated at the 0.05 and 0.001 levels, denoted by *, and ***, respectively.

**Table 6:** Segment-Specific Beliefs about Plant-Based Meat Alternatives Compared to Meat.

Item Description	Total	Cluster 1	Cluster 2	Cluster 3	Cluster 4	F-Value _(3, 1196)_
Plant-based meat substitutes should taste like meat	4.48	4.63 ^ab^	4.84 ^a^	4.31 ^bc^	4.22 ^c^	11.851 ***
Information about the negative environmental impact of eating meat is exaggerated	4.28	4.50 ^b^	3.91 ^a^	4.12 ^a^	4.90 ^b^	21.616 ***
Reducing my meat consumption would not positively impact the environment	4.16	4.32 ^bc^	3.85 ^a^	4.03 ^ab^	4.72 ^c^	15.684 ***
I try to eat as many plant-protein products as possible (e.g., pulses)	4.16	3.82 ^bc^	5.03 ^a^	4.09 ^b^	3.48 ^c^	61.204 ***
I try to eat as many plant-based meat substitutes as possible to reduce my meat intake	3.59	3.08 ^b^	4.59 ^d^	3.66 ^c^	2.59 ^a^	81.579 ***
Plant-based meat substitutes replace meat in my kitchen	3.31	2.71 ^a^	4.31 ^c^	3.38 ^b^	2.42 ^a^	77.548 ***

Values represent mean scores on a 7-point scale, anchored at “totally disagree” and “totally agree”. Different superscripts within rows indicate statistically significant differences between groups at *p* < 0.05 based on one-way ANOVA with Scheffé post hoc tests. Significant differences between clusters are indicated at 0.001 levels with ***.

**Table 7:** Engagement in Sustainable and Ethical Food Consumption Behaviours across Consumer Segments.

Item Description	Total	Cluster 1	Cluster 2	Cluster 3	Cluster 4	F-Value _(3, 1196)_
I try not to throw away food	6.16	6.25 ^b^	6.32 ^b^	5.97 ^a^	6.23 ^ab^	6.989 ***
I do not waste food	6.00	5.99 ^ab^	6.19 ^b^	5.85 ^a^	6.10 ^ab^	4.947 **
I try to use leftovers to make new meals or snacks	5.50	5.32 ^a^	5.85 ^b^	5.37 ^a^	5.47 ^a^	7.866 ***
I choose free-range eggs	5.49	5.44 ^c^	6.13 ^c^	5.33 ^b^	5.03 ^a^	24.698 ***
I avoid purchasing eggs from caged hens	4.99	4.83 ^b^	5.65 ^c^	4.96 ^b^	4.33 ^a^	22.886 ***
I choose locally produced food products	4.88	4.75 ^b^	5.58 ^c^	4.77 ^b^	4.30 ^a^	40.521 ***
I choose foods produced in an environmentally friendly way	4.77	4.58 ^b^	5.74 ^c^	4.67 ^b^	3.88 ^a^	91.162 ***
Whenever possible, I buy fish from sustainable fisheries	4.47	4.23 ^ab^	5.32 ^c^	4.32 ^b^	3.86 ^a^	40.433 ***
I limit my meat consumption	3.97	3.49 ^b^	4.87 ^d^	4.21 ^c^	2.71 ^a^	68.614 ***

Values represent mean scores on a 7-point scale, anchored at “Never” and “Always”. Different superscripts within rows indicate statistically significant differences between groups at *p* < 0.05 based on one-way ANOVA with Scheffé post hoc tests. Significant differences between clusters are indicated at the 0.05, 0.01, and 0.001 levels, denoted by **, and ***, respectively.

**Table 8:** Overview of consumer clusters identified through food-related lifestyle segmentation.

Dimension	Cluster 1—Traditionalists (18.5%)	Cluster 2—Conscious Food Enthusiasts (24.6%)	Cluster 3—Moderates (38.8%)	Cluster 4—Careless Food Lovers (18.2%)
FRL profile	High food involvement but very low innovativeness.	Very high food involvement and innovativeness; highly engaged “foodies”.	Moderate food involvement and openness, striking a balance between tradition and novelty.	High involvement and innovativeness; hedonistic, driven by enjoyment and novelty. Lowest responsibility.
Perception of PBMAs	Rather sceptical about PBMAs’ sensory and nutritional qualities.	Most positive perceptions; PBMAs’ highest ratings for taste, nutritional value, protein completeness and environmental benefits.	Neutral to moderately positive views; ratings close to the total mean for most attributes; neither particularly enthusiastic nor strongly critical.	Negative or indifferent; critical of taste, nutritional value and environmental benefits; highest scepticism regarding the environmental advantages of meat reduction.
PBMA purchasing behaviour	The highest share of non-purchases.	Most frequent buyers.	Rather occasional buyers.	About one-third buy occasionally; 31% never purchase.
Sustainability attitudes	Lower involvement in animal welfare and environmentally oriented purchasing, with moderate responsibility overall.	Highest overall engagement: strong preference for animal welfare, support for local and environmentally friendly production; the highest tendency to limit meat consumption.	Moderate engagement in most sustainability-related practices.	High commitment to avoiding food waste, but consistently lowest scores for animal welfare, environmentally friendly food choices and meat reduction.

## Data Availability

The data presented in this study are available on request from the corresponding author because the data have not yet been made available in publicly available databases.
